# Semantic framework for mapping object-oriented model to semantic web languages

**DOI:** 10.3389/fninf.2015.00003

**Published:** 2015-02-25

**Authors:** Petr Ježek, Roman Mouček

**Affiliations:** ^1^New Technologies for the Information Society, Faculty of Applied Sciences, University of West BohemiaPlzeň, Czech Republic; ^2^Department of Computer Science and Engineering, Faculty of Applied Sciences, University of West BohemiaPlzeň, Czech Republic

**Keywords:** EEG/ERP portal, electrophysiology, object-oriented code, ontology, semantic framework, semantic web

## Abstract

The article deals with and discusses two main approaches in building semantic structures for electrophysiological metadata. It is the use of conventional data structures, repositories, and programming languages on one hand and the use of formal representations of ontologies, known from knowledge representation, such as description logics or semantic web languages on the other hand. Although knowledge engineering offers languages supporting richer semantic means of expression and technological advanced approaches, conventional data structures and repositories are still popular among developers, administrators and users because of their simplicity, overall intelligibility, and lower demands on technical equipment. The choice of conventional data resources and repositories, however, raises the question of how and where to add semantics that cannot be naturally expressed using them. As one of the possible solutions, this semantics can be added into the structures of the programming language that accesses and processes the underlying data. To support this idea we introduced a software prototype that enables its users to add semantically richer expressions into a Java object-oriented code. This approach does not burden users with additional demands on programming environment since reflective Java annotations were used as an entry for these expressions. Moreover, additional semantics need not to be written by the programmer directly to the code, but it can be collected from non-programmers using a graphic user interface. The mapping that allows the transformation of the semantically enriched Java code into the Semantic Web language OWL was proposed and implemented in a library named the Semantic Framework. This approach was validated by the integration of the Semantic Framework in the EEG/ERP Portal and by the subsequent registration of the EEG/ERP Portal in the Neuroscience Information Framework.

## 1. Introduction

Our research group specializes in research of brain electrical activity and operates a laboratory in which the techniques and methods of electroencephalography (EEG) and event related potentials (ERP) are widely used. In addition to the experimental work, which is mostly focused on recording and analysis of cognitive ERP, we are working on the development of the software and hardware infrastructure for research in electrophysiology (Moucek et al., 2014). Our experimental work is typically very time consuming and vast amounts of experimental data are produced at various stages of processing from data recording to their final interpretation. Based on this experience, we know that the means supporting the semantic description of electrophysiological data and the software systems improving the storage, management and sharing of these data (at least within a research group) contribute to long-term understanding of these data and significantly increase research efficiency. This became even more important when we decided to share our data, processing steps and workflows within a wider community.

The subjects of long-term preservation of data, quality and range of their semantic description, and data sharing itself are broadly discussed in the community. A variety of experimental approaches, techniques and methods, targeted subjects, hardware and software infrastructures, etc. used during electrophysiological experiments lead to the accumulation of heterogeneous data in the domain. The semantics of these data means that they are accompanied by metadata that specify their meaning. Metadata can be seen as the links to a specific dictionary, in which the described data are explained and defined, for example, by using a plain text, sets of values or even formal logic. The richness and accuracy of the semantic content of these dictionaries determines the level of semantic description of data. Then the absence of well-defined metadata structures in addition to the absence of standardized and generally used data formats for raw data, are the most pressing difficulties in the domain [as described e.g., in Teeters et al. (2008)].

In the real world electrophysiological data are stored in numerous, often proprietary, data formats and annotated by metadata differently in both range and quality sense. Moreover, a number of sophisticated implementations of data repositories including file systems and databases of different types are available. Then the domain data are also annotated respecting limitations of the used implementations. If we take into account these conceptual and technological heterogeneities and not give up efforts to increase research efficiency in the field by sharing various computational resources such as raw data, metadata, processing methods and workflows among laboratories, then to use a generally readable data format, create a suitable semantic description of data, and develop a tool for the long-term management and sharing of data and metadata in the domain are the first important steps to achieve this complex goal.

However, a higher level of data sharing requires compliance with the minimum standards for domain data and metadata and their structures. The content of these standards and an abstract level of such descriptions are currently broadly discussed in the community (e.g., within INCF Bjaalie and Grillner, 2007 data sharing activities) because a well-specified content and appropriately chosen level of abstraction could bring real sharing opportunities to the domain. On the other hand, one must be careful not to discourage community researchers to provide data and metadata in some standardized formats and structures by introducing too abstract semantic descriptions to them. Although this topic and standardization efforts themselves are very important for the future of data sharing in the domain, their more extensive discussion is out of scope of this article. However, it is important to introduce them since they are closely related to a more specific task (Section 2.3), the description of which forms the core of this article.

Any proposed abstract level of the semantic description of domain metadata and their relationships has to be accompanied by selecting expressive means and subsequently by selecting technologies that promote data sharing. Currently there are two main approaches for building semantic structures for metadata. The first approach is to use conventional data models and structures and conventional programming languages providing access to data. The second approach assumes the use of formal representations of ontologies, known from the knowledge representation field, such as description logic or Semantic Web languages and (eventually) reasoning provided by software agents. Discussions of these approaches and their practical applications have been held for decades. They related mainly to expert systems in the past; currently difficulties and perspectives of the Semantic Web languages and technologies are mostly discussed.

The initial concept of the Semantic Web, as was introduced in Berners-Lee et al. (2001), was based on artificial intelligence techniques; knowledge bases accessed via web interfaces served for automatic reasoning provided by software agents. However, this approach has been continuously changing since the years 2004–2005 and the Semantic Web started to be more viewed as a large distributed database. This perspective has two following fundamental aspects. The first one is known as the knowledge acquisition bottleneck; acquisition of knowledge is a time consuming process that is made usually by domain experts. As a result, knowledge bases are rather small comparing to conventional data repositories. This bottleneck is currently even more visible in the context of big data collected from the increasing number and due to technical capabilities of hardware acquisition devices. The second aspect is related to the processing of conventional data that in most cases does not mean producing entirely new data by automated reasoning. Data are supposed to be just queried and linked to other data. This view of data is one of the key concepts of linked data (Berners-Lee, 2006).

The Semantic Web expresses data by a triple-oriented language, Resource Description Framework (RDF) (Manola and Miller, 2004). When communities working in knowledge representation and web engineering started to interact more, there was a question if XML as a hierarchically oriented language or RDF as a language supporting graph structures is more suitable for the representation of meaning. Finally, XML is used as a means for the serialization of RDF graphs. RDF/XML syntax as the first standardized RDF syntax is still widely used. Because expressivity of RDF is limited, W3C^1^ defined a more powerful language with more capabilities for expressing meaning, Web Ontology Language (OWL) (Dean and Schreiber, 2004). While RDF was accepted by a larger community, the OWL language was a large burden for the practical application of the Semantic Web. This language, based on description logic, is usually unintelligible to non-experts. Moreover, not many OWL constructions have been really used in real applications yet. To cope with these difficulties, specific OWL dialects supporting different aspect of the resulting semantic model, are currently available. The aims of the subsequent OWL2 specification are to improve datatype expressivity, provide better organization of imports, and remove difficulties with different versions of OWL syntaxes.

The concept of linked data is currently one of four ways to expose RDF data on the web. Another way is based on providing SPARQL endpoints to explore data using SPARQL queries (an example is the RDF platform at The European Bioinformatics Institute, 2014). SPARQL (Prud'hommeaux and Seaborne, 2008) supported by W3C is the most spread standardized language for queering RDF graphs. Other alternatives include publishing data directly to the web as dump files by using one of the serialization formats for RDF graphs and using RDFa (Adida et al., 2013) for expressing structured data in a markup language (e.g., HTML). The first two approaches (linked data, SPARQL endpoints) are technologically advanced, give users more opportunities to work with data, but they require specific knowledge of developers and administrators and place higher requirements on hardware equipment. There is also a difficulty with overall availability of SPARQL endpoints; the statistics is provided by Open Knowledge Foundation (2014). As a result, these advanced models serve mainly for initial exploring the contents of the data, while in normal use the data are downloaded (as dump files) and processed locally.

Domain ontologies have been playing a significant role in information systems for a long time (Chandrasekaran et al., 1999) and they are well-designed for heterogeneous data description. In the past ontologies were mainly created independently, they did not cope with vast amounts of data and focused on logical reasoning. This approach has been changing together with the changing view of the Semantic Web. Currently, ontologies are created using a bottom-up strategy to take advantage of already existing data. These ontologies are then covered by upper-level ontologies or ontological background models. Since for a newly created dataset we hardly find a comprehensive ontology, there are two basic options (which can be used in parallel), to create an ontology for it. In the first approach, it is supposed to find relevant types of objects and relations in already existing ontologies, compare them, find the most suitable types and reuse them. In the second approach, new types are defined, collected and organized in a newly designed ontology. The mixed strategy, when the common types are reused from other ontologies and specific types are newly defined, is for example used during the development of the Ontology for Experimental Neurophysiology (OEN) (Le Franc et al., 2014).

In the electrophysiology domain at least the following initiatives are worth briefly describing. Ontology for Biomedical Investigations (OBI) (Brinkman et al., 2010) is an ontology for biological and clinical investigation description. Its terminology contains domain-specific terms and universal terms for general biological and technical usage. It uses OWL as a formal language. NEMO (Dou et al., 2007) is an ontology describing EEG, averaged EEG (ERPs), and ERP data analysis results; it lacks possibilities to describe experimental protocols and restrictions. odML (Grewe et al., 2011) is an open, flexible, easy-to-use and unrestricted transporting format for annotation and sharing of electrophysiology data that can be implemented into any recording or management tool. The odML model for metadata defines four entities (Property, Section, Value, RootSection). EEG/ERP Portal (EEGBase) (Jezek and Moucek, 2012) uses a classic relational database and object-oriented structures to create the domain model.

Looking at the semantic expressivity of ontologies from the point of view of automated processing, the higher the level of formalization is, the easier it is to use the ontology for sharing and reasoning, since it is more machine-processable. On the other hand, it is difficult to develop such an ontology. Moreover, the use of formalisms with high expressivity leads to difficulties with decidability and computational complexity when reasoning. Then these formalisms have to be limited in their expressivity to ensure that the resulting ontologies are usable in practice for automated reasoning. Looking at the present state, then despite the increasing popularity of storing data in semantic repositories (which are based on flexible physical data models such as graphs) and retrieving them by using languages providing higher sematic expressivity, most data are still stored in conventional repositories such as files and relational databases [a list of DBMS ranked by their current popularity is available in Solid IT (2014)]. Taken into account big conventional data collected from a number of hardware sensors, familiarity of large groups of developers, administrators and users with conventional data repositories, and simplicity of publishing RDF data (transformed from conventional data) as dump files, we can hardly anticipate a substantial change in the use of current types of repositories in the near future.

Shared conventional data have often read-only access to third party subjects. It means that only data owners are entitled to add semantics to them. Moreover, conventional relational data repositories due to their limitations in semantic expressivity naturally exclude to add more complex semantic information to data. Nevertheless, richer semantic descriptions can be still added later. This can be done by transforming a conventional repository to a semantic repository (e.g., to RDF triple stores) or by semantic enrichment of the conventional programming language that provides access to the data stored in conventional repositories. However, the first approach requires using the SPARQL language for later data access and retrieval. Then to avoid using the Semantic Web languages and technologies to the last moment and still to cope with opportunities to add richer semantic descriptions to data, it is necessary to semantically enrich a conventional programming language.

The rest of the article is organized in the following way. The Section Materials and Methods contains the brief description of commonly used data models and languages both in software engineering and knowledge representation fields, the state of the art in the mapping between these two approaches, and the core of the article - the Semantic Framework for the mapping an object-oriented model to semantic web languages. The Section Results provides information about the performance and experimental evaluation of the Semantic Framework. The Section Discussion mainly deals with the future development of the Semantic Web and related methods and technologies for data sharing.

## 2. Materials and methods

### 2.1. Data models and languages

Analyzing the use of conventional semantic data models (Biller and Neuhold, 1977; Simsion and Witt, 2004), essentially the two following data modeling formalisms are widely used: the entity-relation (ERA) model and object-oriented (OO) model. Newer formalisms, e.g., the Enhanced-entity–relationship (EER) model, only combine these two basic formalisms. The Unified Modeling Language (UML) is the most used language for modeling an application structure, behavior, architecture, business processes, and data structures in classic software engineering. A UML model consists of three major categories (classifiers, events, behaviors) of model elements, each of which may be used to make statements about different kinds of individual things within the system being modeled (Bock et al., 2013).

The models defining object types and relations in knowledge engineering are connected with the development of ontologies. Within the Semantic Web languages, RDF is a standard model for data interchange, RDFS is a language for representing simple RDF vocabularies on the Web, and OWL as a computational logic-based language represents rich and complex knowledge about things, groups of things, and relations between things (Dean and Schreiber, 2004). However, the Semantic Web languages and technologies have not been popular for developing application programs (Antoniou and van Harmelen, 2004). Within the classic software engineering discipline the popularity of script-based languages in neuroinformatics has been continuously rising (Garcia and Fourcaud-Trocm, 2009), but these languages have not been considered to be suitable for the development of large systems (Scott, 2004). Thus, an object-oriented system is still the first choice when we want to design and implement a large, robust and reliable software system.

There is a question how we can use advantages of both the models used in software engineering and knowledge representation disciplines and how we can construct a mapping between them if this is possible. In general, Semantic Web languages associate three types of features used in the object-oriented world. They describe reality on the conceptual level independent of technological restrictions, i.e., they are similar to UML representations. They also constitute a database schema for the base of facts (RDF). Eventually they are processed by software tools in the implemented application, i.e., they are part of the implementation.

It is very difficult to compare UML and OWL. Although both are languages for modeling and have several structural similarities, they have different capabilities and different approach to semantics. They both have classes, instances, inheritance, enable defining cardinality restrictions, etc. On the other hand, OWL classes are viewed like labels for concepts, while UML classes are viewed like templates for instances. The most substantial differences deal with the meaning of instances (individuals in OWL) and properties. In UML any class is an object that can be instantiated. This process has its semantics like assigning values to attributes. Moreover, instances have a run time semantics. In OWL a class is a category, no instantiation process is defined. OWL individuals, identifiers for domain things, are defined independently of classes. If an OWL individual meets the criteria for the class membership, then it is a member of the class. It has no state, storage or runtime semantics. UML properties always belong to a class, while OWL properties are stand-alone entities. OWL properties are double types; object and datatype properties. The first one links an individual to an individual and the second one links individuals to data values. Understanding of a class extent is also different. While UML classes work inside a program where they are defined, OWL classes provide features to share classes among domains. OWL classes may be linked to a list of class descriptions (*Intersection, Union, Complement*). A property restriction, a special kind of class description, describes an anonymous class, namely a class of all individuals that satisfy the restriction. OWL distinguishes two kinds of property restrictions: value constraints (e.g., *allValuesFrom, someValuesFrom*) and cardinality constraints Dean and Schreiber (2004). OWL can discipline names using *AllDifferent*, *SameAs* or *DifferentFrom* constructs.

**Figure 1:**
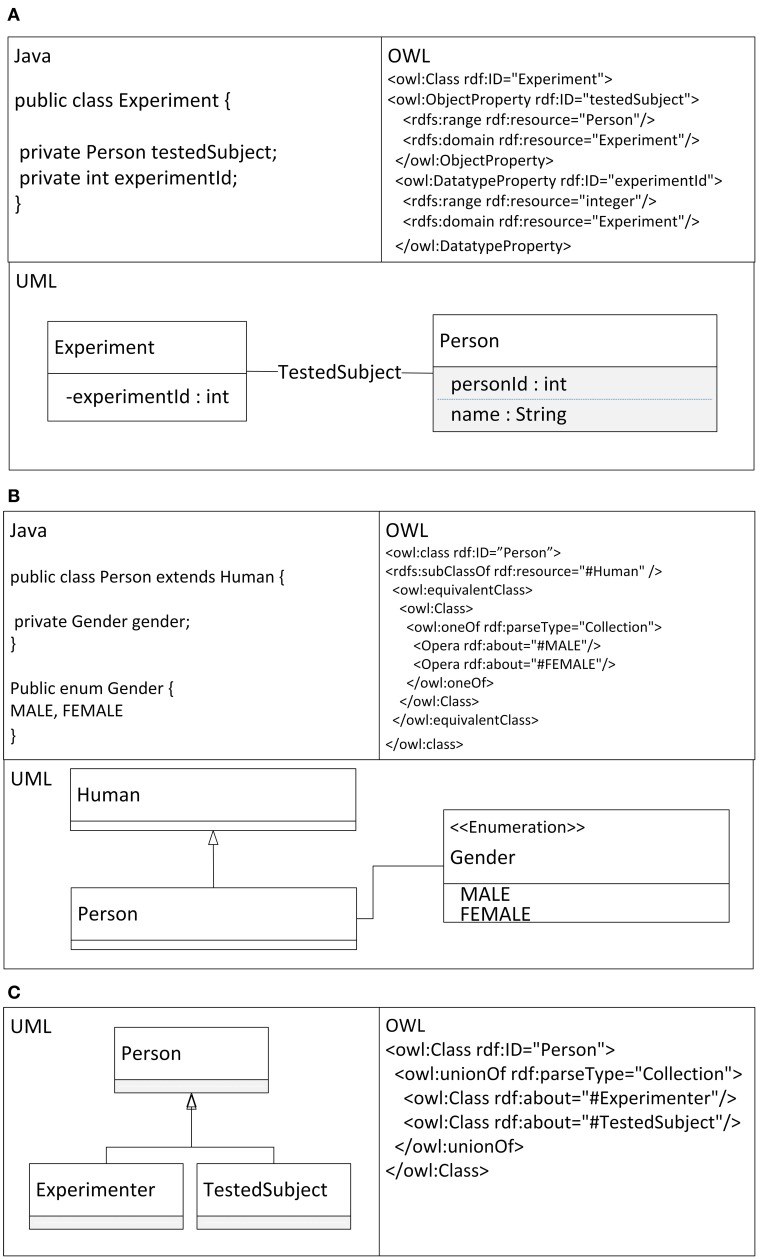
**Practical examples of Java code, OWL, and UML Features. (A)** Definitions of classes with primitive and object properties. **(B)** Definitions of inheritance and enumeration. **(C)** Definitions of union classes.

ODM (Ontology Definition Metamodel) (Object Management Group, 2009) describes the relationships between the relevant features of UML and OWL in detail. Described differences are summarized in Table 1 and shown together with a relevant Java code in Figure 1.

**Table 1:** **UML, OWL and Java Features Comparision**.

**UML**	**OWL**	**Java**	**Comment**
Class, atomic type, property ownedAttribute	owl:Class	*Class*	
Instance	Individual	Class instance	OWL owl:individual class independent
Owned attribute, association	owl:DataTypeProperty, owl:ObjectProperty	Class attributes: primitive data types/objects	OWL has only global attributes
Subclass, generalization	owl:subclass, owl:subproperty	*Extends*, inherited classes and properties	Java does not support multiple inheritance
Enumeration	owl:oneOf	*Enum*	
Disjoint	owl:disjointWith, owl:unionOf	One object always an instance of exactly one class, but we should pay attention to class inheritance	
Multiplicity	owl:MinCardinality, owl:MaxCardinality	–	
Package	Ontology	*Package*	
Dependency	RDF:property	Methods parameters or return value	
–	owl:intersectionOf, owl:unionOf, owl:complementOf, owl:DifferentFrom, owl:AllDifferentFrom, owl:allValuesFrom, owl:someValuesFrom, owl:SameAs	–	

### 2.2. Related work

Focusing on conventional data resources and programming tools, specifically a relational database and an object-oriented code, we briefly describe several approaches and tools that map a relational schema or an object-oriented code to the Semantic Web languages. Some of these approaches exist only as initial proposals or prototypes published in scientific papers, while some of them have been really implemented as available frameworks.

An RDF triple might be represented as a row in a table of relational database. This table has two columns, corresponding to the subject and the object of the RDF triple. The name of the table corresponds to the predicate of the RDF triple. The D2RQ (Bizer and Seaborne, 2004) platform is a system for accessing relational databases as virtual, read-only RDF graph. It uses a declarative language to describe a mapping between a relational database schema and RDF; the content of relational database is not replicated into an RDF store. The D2RQ platform provides, for example, possibilities to query a non-RDF database using the SparQL (Prud'hommeaux and Seaborne, 2008) query language, to create custom dumps of the database in RDF formats, and to access information in a non-RDF database using the Apache Jena API. METAMorphoses (Švihla, 2007) is a data transformation processor from a relational database into RDF. An XML template document defines a set of mapping rules and queries for obtaining data stored in a relational database.

There are approaches and tools that provide limited possibilities to map common syntaxes of an object-oriented code to an OWL representation. These tools map fundamental OWL features, it means that only the basic semantic expressivity of OWL is used.

A mapping of OWL classes to Java Interfaces is described in Kalyanpur et al. (2004). The mapping to a Java Interface instead of a common Java class enables the expression of multiple inheritance of OWL properties. The back transformation is described in Koide et al. (2005), where the OWL processor SWCLOS3, which is at the top of the Common Lisp Object System (CLOS), is described. Whereas CLOS allows lisp programmers to develop object-oriented systems, SWCLOSS allows programmers to construct domain and task ontologies in software application fields. Java2OWL-S (Ohlbach, 2012) is a tool which is able to generate OWL directly from JavaBeans. It uses two transformations. The first transformation is from JavaBeans into WSDL (Web Service Description Language). The input of this transformation is formed by a Java class and the output is a temporary WSDL file. The second transformation generates OWL from the WSDL file. Concerning one-side transformations (from conventional languages to Semantic Web languages), these tools with common semantic expressivity work quite satisfactorily because the semantic expressivity of the object-oriented code is lower than the semantic expressivity of the OWL language. However, in these tools, no possibility to enrich the object-oriented code with additional semantic constructs exists.

The Semantic Object Framework (SOF) (Po-Huan et al., 2009) utilizes embedded comments in source codes to describe semantic relationships between classes and attributes. The eClass (Liu et al., 2007) is a solution that changes Java syntax to embed semantic descriptions into an object-oriented source code. These frameworks thus enrich the input object-oriented code with additional semantics using but their use is difficult because they require a modified compiler and Java interpreter.

### 2.3. Semantic framework

Drawbacks and limits of the tested frameworks motivated us to introduce a software prototype that allows its users to add additional semantics directly into the Java object-oriented code. The Java code works with conventional data repositories. A mapping that allows transformation of this code into the Semantic Web language OWL was proposed and implemented as a library, the Semantic Framework. This solution is usable by software engineers and not only by experts in the Semantic Web. It serves a community of developers/researchers that develop/use object-oriented systems and need to provide an output in OWL. This approach also does not burden users with additional demands on programming environment since we use reflective Java annotations (metadata added to the Java source code and retrieved at run-time) as standard syntactic structures in Java. Moreover, additional semantics even need not to be written by the programmer directly to the code, but it can be collected from non-programmers using a graphic user interface. The presented approach is further discussed from performance (Section 3.1) and usability (Section 3.2) perspectives. It was validated by the integration of the Semantic Framework in the EEG/ERP Portal, together with its registration in the Neuroscience Information Framework (NIF).

### 2.4. Javabean to OWL mapping

Java stores data in JavaBeans^2^, often called Plain Old Java Objects (POJOs). The transformation of JavaBean representation into an OWL ontology is described in Definition 1.

**Definition 1**. *(Extraction process from a JavaBean structure)*

The process is the transformation of a set of JavaBeans J to an ontology O that satisfies:

∀ *J*_i_ ∃ *OC*_i_ ∈ O; *OC*_i_ is an OWL class; i ∈ {1..n}; n is the number of JavaBeans.

∀ *J*_i_ that is a superclass of *J*_j_ ∃ *OC*_i_ that is a superclass of *OC*_j_ ∈ O; *OC* is an OWL class; i ∈ {1..n}; n is the number of JavaBeans; j ∈ {1..m}; m is the number of *J*_i_ subclasses.

∀ *Jf*_j_ ∃ *OC*_i_ ∈ O; its *OC*_i_ extent is a DataType property ∈ O ⇔ *Jf*_j_ is an atomic type field of JavaBean; *OC*_i_ is an OWL class; *Jf*_j_ is a field of JavaBean; i ∈ {1..n}; n is the number of OWL classes; j ∈ {1..m}; m is the number of *J*_i_ fields.

∀ *Jf*_j_ ∃ *OC*_i_ ∈ O; its *OC*_i_ extent is an Object property ⇔ *Jf*_j_ is an object type field of JavaBean; i ∈ {1..n}; n is the number of OWL classes; j ∈ {1..m}; m is the number of JavaBean fields.

∀ *J*_inst_ of *J*_i_ ∧ ∀ *Jf*_ij_ ∃ *OL*_ij_ ∈ O so that *OL*_ij_ ≃ *Jf*_ij_; *J*_inst_ is an instance of *J*_i_; *Jf*_ij_ is a field of *J*_i_; i ∈ {1..n}; n is the number of JavaBeans; j ∈ {1..m}; m is the number of *J*_i_ fields; *OL*_ij_ is an OWL literal.

An example of a mapping of a JavaBean to an OWL construct is shown in Listing 1.1. The JavaBean *Person* has two attributes, *researchGroups* and *firstname*. The first one is an association relation to the *ResearchGroup* class, while the second one is an atomic type. Get/set methods are omitted to keep readability. Listing 1.2 shows a fundamental serialization of this class into an OWL structure. The attribute *firstname* is serialized to a *DataTypeProperty* while the attribute *researchGroups* is serialized to an *ObjectProperty*.

**Listing 1:** **JavaBean Person**.

package cz.zcu.kiv; public class Person { @Id private int id; private String firstname; private List<ResearchGroup> researchGroups; }

**Listing 2:** **OWL Individual**.

<owl:Class rdf:ID=“Person”> <semantic:javaclass>cz.zcu.kiv.Person </semantic:javaclass> </owl:Class> <owl:Class rdf:ID=“ResearchGroup”> <semantic:javaclass>cz.zcu.kiv. ResearchGroup</semantic:javaclass> </owl:Class> <owl:ObjectProperty rdf:ID=“researchGroups”> <rdfs:domain rdf:resource=“&this;Person”/> <rdfs:range rdf:resource=“&this; ResearchGroup”/> </owl:ObjectProperty> <owl:DatatypeProperty rdf:ID=“firstname”> <rdfs:domain rdf:resource=“&this;Person”/> <rdfs:range rdf:resource=“&xsd;string”/> </owl:DatatypeProperty> <owl:DatatypeProperty rdf:ID=“id”> <rdfs:domain rdf:resource=“&this;Person”/> <rdfs:range rdf:resource=“&xsd;integer”/> </owl:DatatypeProperty>

Although the described mapping works quite satisfactorily, OWL concepts described in Section 2.1 are not covered. When we want to use more capabilities of OWL, we have to enrich the object-oriented code with additional semantic expressions. Looking for a suitable way to extend a current object-oriented code, we decided to pursue a preliminary idea (Jezek and Moucek, 2011b) based on using Java Annotations (MicroSystems, 2008).

Java Annotations have several benefits. Firstly, they can be added, as a special form of syntactic metadata, to a Java source code. Secondly, they are reflective, i.e., they can be embedded within the compiled code and retrieved at runtime. Moreover, Java Annotations are a part of the Standard Java Development Kit; they can be processed immediately using Java 5.0 or higher. Finally, Java Annotations are used in current software development (by several common frameworks, e.g., Spring, Hibernate, Java Persistent API); hence, software developers can work with this extension without difficulties.

The theoretical extraction of JavaBeans annotations and their transformations to OWL documents is formally described in Definition 2.

**Definition 2**. *(Java annotation extraction process)*

The process is the transformation of a set of Java annotations JA to a resources R in the ontology O that satisfies:

∀ *JA*_i_ ∈ class annotations ∃ OWL class *R*_i_ ∈ O; i ∈ {1..n}; n is number of Java class annotations.

∀ *JA*_i_ ∈ property annotations ∃ OWL property *R*_i_ ∈ O; i ∈ {1..n}; n is number of Java property annotations.

An example of using annotations is given in Listing 1.3. The class *Person* has attributes *firstname* and *researchGroups* as defined in Listing 1.1. Moreover, the attribute *dateofBirth* is added. The *firstname* attribute is defined with a value that is get from the value constraint *GivenNames* by using the *@SomeValuesFrom* annotation. The attribute *dateofBirth* is defined with cardinality equal to 1 and the attribute *researchGroups* is defined with minimum cardinality equal to 1. In addition, the class *Person* is defined as equivalent to the class *TestedSubject* using the annotation *EquivalentClass*. The serialization of this JavaBean is shown in Listing 1.4. The class *Person* is a subclass of *owl:cardinality*, *owl:someValuesFrom* and *owl:minCardinality* OWL restrictions.

**Listing 3:** **Annotated Java Bean**.

package cz.zcu.kiv; @EquivalentClass (“http://cz.zcu.kiv/TestedSubject”) public class Person { @Id private int id; @SomeValuesFrom(stringValues = “http://cz.zcu.kiv/GivenNames”) private String firstname; @Cardinality(1) private Date dateofBirth; @MinCardinality(1) private List<ResearchGroup> researchGroups;

**Listing 4:** **OWL Serialization of Annotated Java Bean**.

<owl:Class rdf:ID=“Person”> <owl:equivalentClass> <owl:Class rdf: about=“http://cz.zcu.kiv/ TestedSubject”/> </owl:equivalentClass> <rdfs:subClassOf> <owl:Restriction> <owl:cardinality rdf:datatype= “&xsd;int“>1</owl:cardinality> <owl:onProperty> <owl:DatatypeProperty rdf: ID=“dateofBirth”/> </owl:onProperty> </owl:Restriction> </rdfs:subClassOf> <rdfs:subClassOf> <owl:Restriction> <owl:someValuesFrom> <owl:DataRange> <owl:oneOf rdf:parseType=“Resource”> <rdf:rest rdf:resource=“&rdf;nil”/> <rdf:first rdf:datatype=“&xsd; string”> http://cz.zcu.kiv/GivenNames </rdf:first> </owl:oneOf> </owl:DataRange> </owl:someValuesFrom> <owl:onProperty> <owl:DatatypeProperty rdf: ID=“firstname”/> </owl:onProperty> </owl:Restriction> </rdfs:subClassOf> <rdfs:subClassOf> <owl:Restriction> <owl:minCardinality rdf: datatype=“&xsd;int”>1 </owl:minCardinality> <owl:onProperty> <owl:ObjectProperty rdf: ID=“researchGroups”/> </owl:onProperty> </owl:Restriction> </rdfs:subClassOf> <semantic:javaclass> cz.zcu.kiv.Person </semantic:javaclass> </owl:Class>

We chose the concepts that have a class and/or property extent (Jezek and Moucek, 2011a) and defined a set of annotations with their mapping to corresponding OWL constructs (Table 2). Most of the proposed annotations are parameterizable. Parameter values shown in Table 2 are examples; they can be changed according to the needs of a specific domain.

**Table 2:** **OWL Mapping of Java Annotations**.

**Java Annotation**	**OWL construct**
@EquivalentClass	<owl:equivalentClass rdf:resource=“http://www.kiv.zcu.cz/Person”/>
(“http://www.kiv.zcu.cz/Person”)	
@EquivalentProperty	<owl:equivalentProperty rdf:resource=“http://www.kiv.zcu.cz/first_name”/>
(“http://www.kiv.zcu.cz/first_name”)	
@Symmetric	<rdf:type rdf:resource=“http:/www.w3.org/2002/07/owl#SymmetricProperty”/>
@Inverse	<owl:inverseOf rdf:resource=“http://www.kiv.zcu.cz/givenname”/>
(“http://www.kiv.zcu.cz/givenname”)	
@AllValuesFrom	<owl:allValuesFrom rdf:resource=“http://www.kiv.zcu.cz/#Persons”/>
(“http://www.kiv.zcu.cz/#Persons”)	
@Transitive	<rdf:type rdf:resource= “http://www.w3.org/2002/07/owl#TransitiveProperty”/>
@AllDifferent	<rdf:type rdf:resource=“http://www.kiv.zcu.cz/#AllDifferent”/>
(“http://www.kiv.zcu/Experiment”)	
@DifferentFrom	<owl:differentFrom rdf:resource=“http://www.kiv.zcu/Experiment”/>
(“http://www.kiv.zcu.cz/Experiment”)	
@SameAs	<owl:sameAs rdf:resource=“http://www.kiv.zcu/Experiment”/>
(“http://www.kiv.zcu.cz/Experiment”)	
@Cardinality(1)	<owl:cardinality rdf:datatype=“http://www.w3.org/2001/XMLSchema#int”>1</owl:cardinality>
@MaxCardinality(1)	<owl:maxCardinality rdf:datatype=“http://www.w3.org/2001/XMLSchema#int”>1</owl:maxCardinality>
@MinCardinality(1)	<owl:minCardinality rdf:datatype=“http://www.w3.org/2001/XMLSchema#int”>1</owl:minCardinality>
@SomeValuesFrom	<owl:someValuesFrom rdf:resource=“http://www.kiv.zcu/Person”/>
(“http://www.kiv.zcu/Person”)	

**Figure 2:**
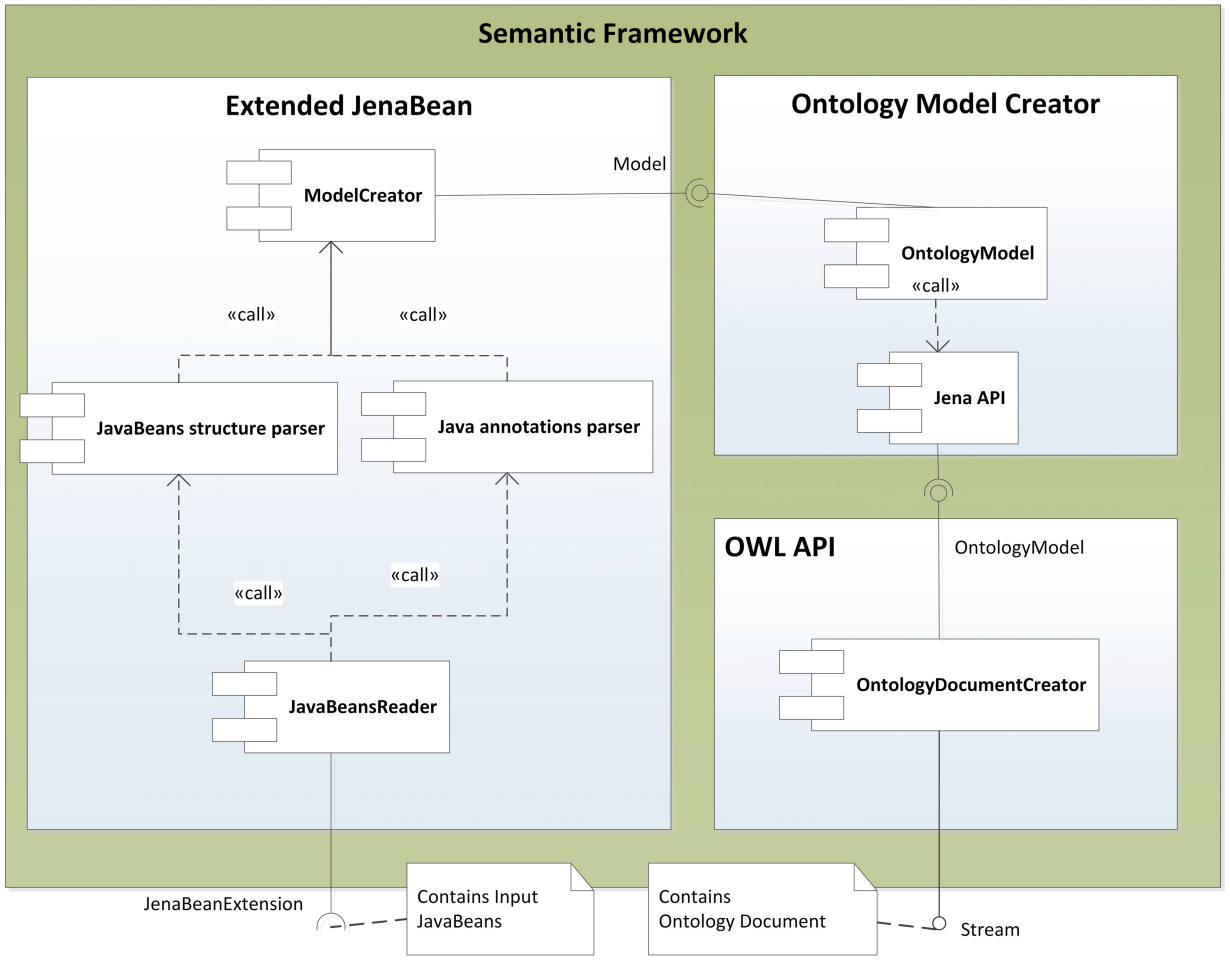
**Component Diagram of the Semantic Framework.** The Semantic Framework reads a list of input JavaBeans using an implemented reader based on Java Reflection API (SUN, 2006). This list passes through two parsers. The first one reads a JavaBean structure; the second one reads supplemented annotations. The parsers create an internal JenaBean representation. This representation is read by Jena API, which provides an ontology model processed by an OWL API. The OWL API implements existing OWL syntaxes and provides methods for serializing the ontology model.

**Figure 3:**
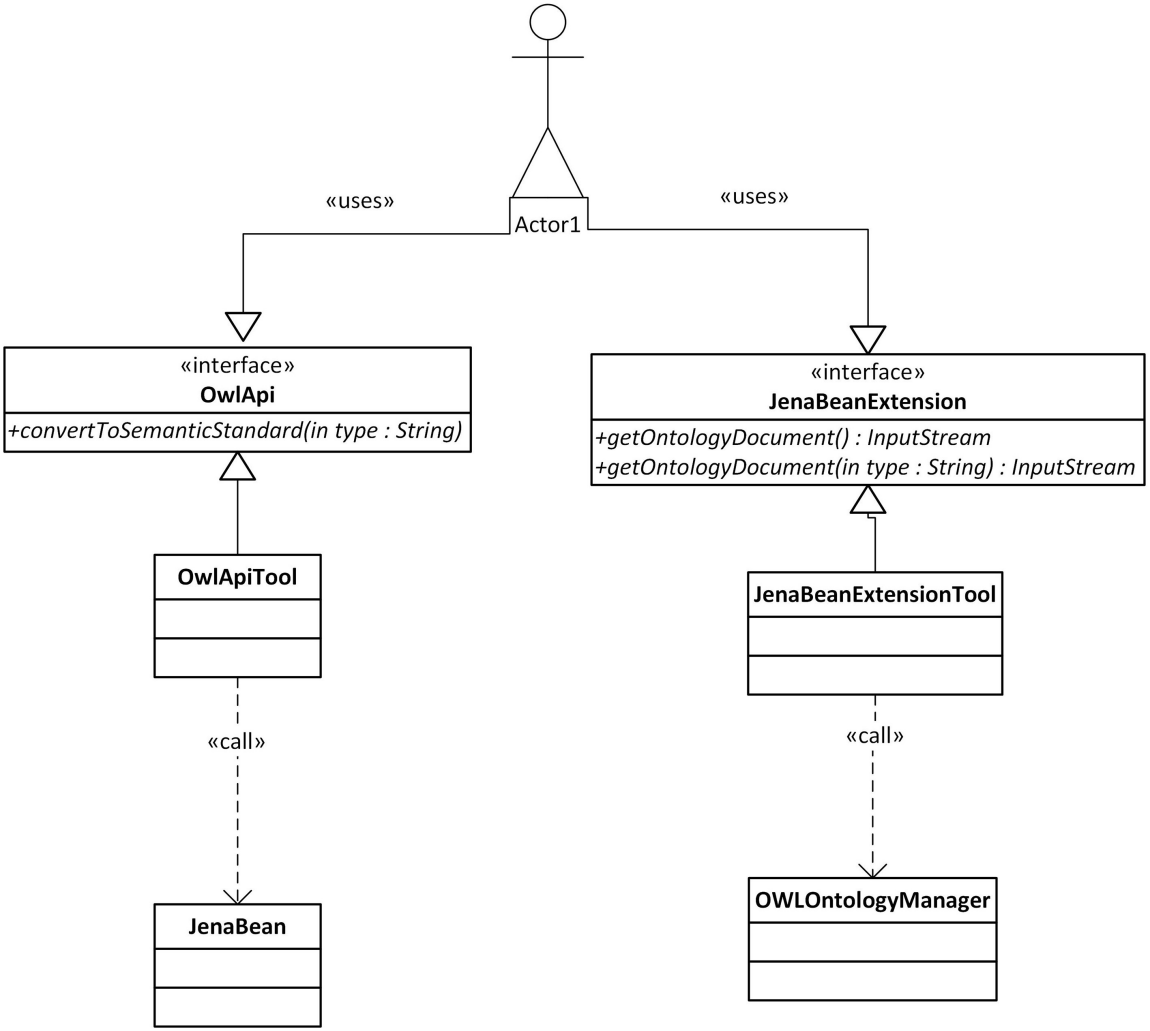
**Class Diagram of the Semantic Framework Interface.** Actor1 represents a client program. The client uses the interface *JenaBeanExtension* having the method *getOntologyDocument* that returns an ontology document in a stream. The returned stream can be serialized into supported formats by an *OwlApi* interface calling the *convertToSemanticStandard* method.

The described mapping was implemented as a library named the Semantic Framework^3^. It processes a set of JavaBeans as an input and produces an ontology document as an output. We did not implement the Semantic Framework from scratch, but extended and integrated already existing tools. The core of the system is extended JenaBean (JenaBean Team, 2010) that enables binding of common JavaBeans to RDF/OWL classes and properties. It internally uses the Jena Framework (Apache Jena Project Team, 2011) with Jena RDF/OWL API to persist JavaBeans. Figure 2 shows the component diagram of the Semantic Framework. The first subcomponent is the extended JenaBean that reads and parses JavaBeans and related Java annotations. The output of the *Extended JenaBean* component is an internal JenaBean model that is transferred to the second, *Ontology Model Creator*, subcomponent. This subcomponent creates an ontology model using an *Ontology Model Factory* and *Jena API* methods. This ontology model extends access to the statements in a RDF data collection by adding support for constructs that are expected to be in an ontology. However, all of the state information is still encoded as RDF triples and stored in the RDF model. The resulting ontology model (in the form of an ontology document) is further processed by the last subcomponent, *OWL API* (Horridge and Bechhofer, 2011), which provides the ontology model in a required serialization format. The UML diagram describing the usage of the Semantic Framework is available in Figure 3.

## 3. Results

### 3.1. Performance evaluation

The time complexity of the Semantic Framework library was tested in the following way. Firstly, we prepared a set of instances of the class *Experiment*. Then, we assigned instances of the classes *Person*, *Scenario*, *Hardware*, and *Data* to each instance of the class *Experiment*. The class *Person* was extended by the set of supported annotations from Table 2. The performance tests were run 10 times and the result was calculated as an average of all program runs. The time complexity of the transformational process was linear with respect to the number of instances. All the tested syntaxes are functionally equivalent; they differ only in the format of the serialized output document (Beckett, 2004).

### 3.2. Experimental evaluation

We defined a simple ontology describing the experimental work in our laboratory. Semantically, it is a modified subset of the NEMO ontology with added terms describing an experimental protocol and restrictions during an experimental session. The ontology structure corresponds to metadata collected during experiments. These metadata are divided into several semantic groups:

- Activity - describes a predefined experimental protocol. It includes information about audio and/or video stimulation, instructions given to tested subjects, detailed descriptions of stimuli (target vs. non-target, timing), etc.
- Environment - describes surrounding conditions such as weather, daytime or room temperature.
- Tested subject - includes information about the tested subject such as laterality, education, age, gender, diseases, and disability.
- Hardware equipment - describes e.g., the type, producer, and serial number of the hardware used.
- Software equipment - describes software used during the experiment. It includes e.g., the name of the software, version, manufacturer, and configuration files if they are used.
- Used electrodes - describes the type, impedance, location, used system, and fixation of the electrodes.
- Data digitalization - describes a set of parameters that influence conversion of data using a specific analog-digital converter. It includes filtration, sampling frequency, and band-pass.
- Signal analysis - describes basic analytic steps during the EEG/ERP signal processing. It includes the determination of the length of the pre- and post-stimulus part of the signal, number of epochs, and text description of the signal-processing procedure.
- Data presentation - describes experimental results or assumptions needed to reproduce an experiment. It includes averaged ERP waves (images of averaged waves), grand averages (images of grand averages), evolution of the ERP signal in time and space (images showing the ERP signal propagation over the scalp), waves description (description of well-known or new waves formed during the study), and link to raw experimental data.
- Signal artifact - contains information describing a compensation method that prevents formation of artifacts. When a method for removing artifacts is used, its description is also placed there. When some artifact totally degrades the signal, the experimenter can define conditions when it is possible to assume that the signal is totally useless.

This simple ontology was built within the development of the EEG/ERP Portal (EEGBase) (Jezek and Moucek, 2012), which is a web application (Neuroinformatics group, University of West Bohemia, 2014) for the storage, long-term management, and sharing of electrophysiology data. The data layer of the EEG/ERP Portal is implemented using a relational database (Oracle 11 g) and POJOs. An object-relational mapping (ORM) is ensured by the Hibernate framework (Bauer and King, 2006). The internal structure (classes and their relationships, annotations) of the data layer is implemented according to the defined ontology. The application layer was developed using the Spring Framework; the presentation layer uses Apache Wicket. Upload of data and metadata is ensured via a set of predefined web forms.

**Figure 4:**
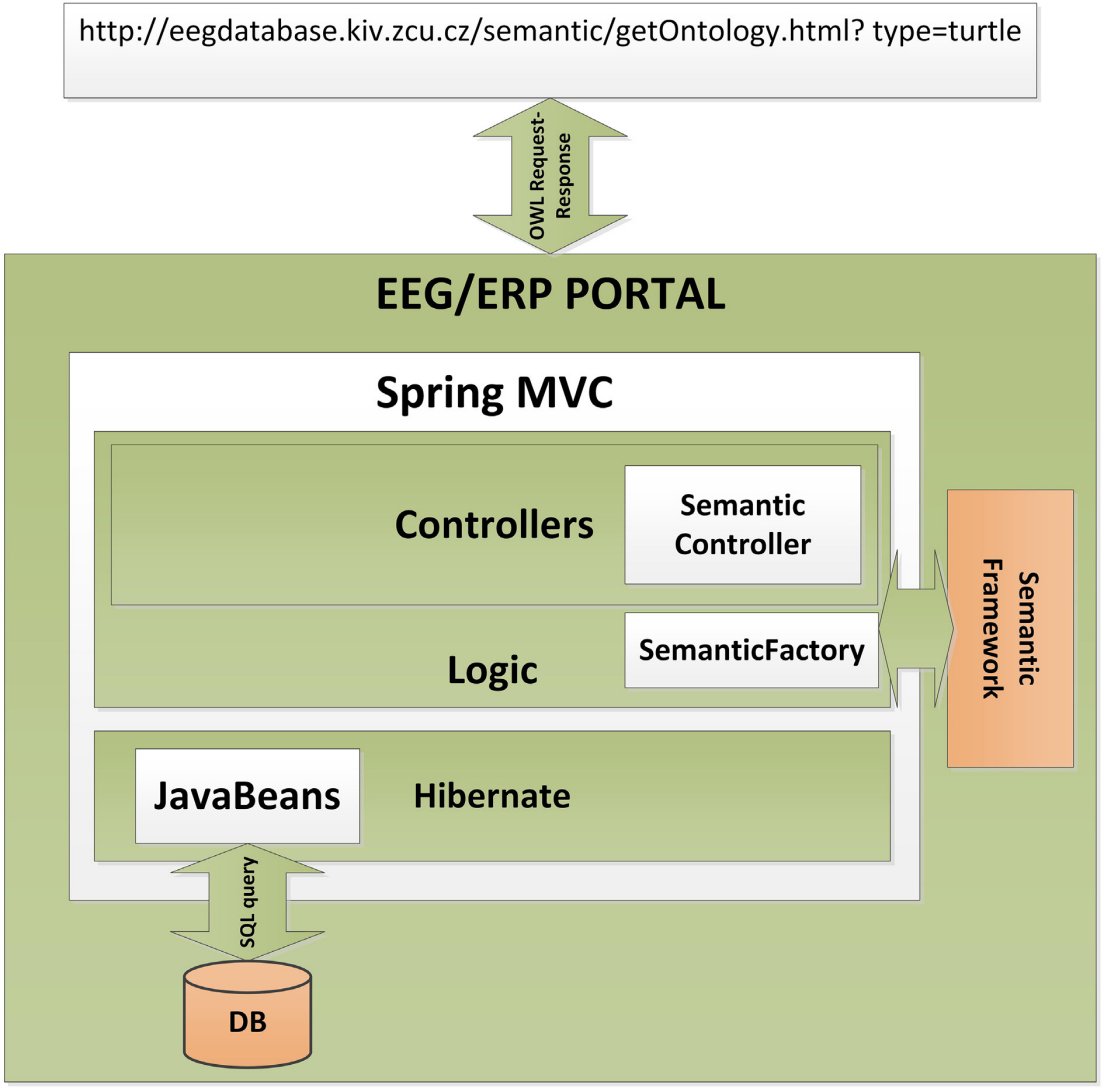
**Integration of the Semantic Framework in the EEG/ERP Portal.** The EEG/ERP Portal is a layered architecture designed according to a Model-View-Controller design pattern. The data layer obtains Javabeans from a relational database. The application layer is controlled by the set of *Controllers* which process user requests originated from a web browser. The specific controller (*Semantic Controller*) processes ontology document requests. This controller calls the integrated Semantic Framework through *Semantic Factory* and returns a serialized ontology document to the user's web browser.

**Figure 5:**
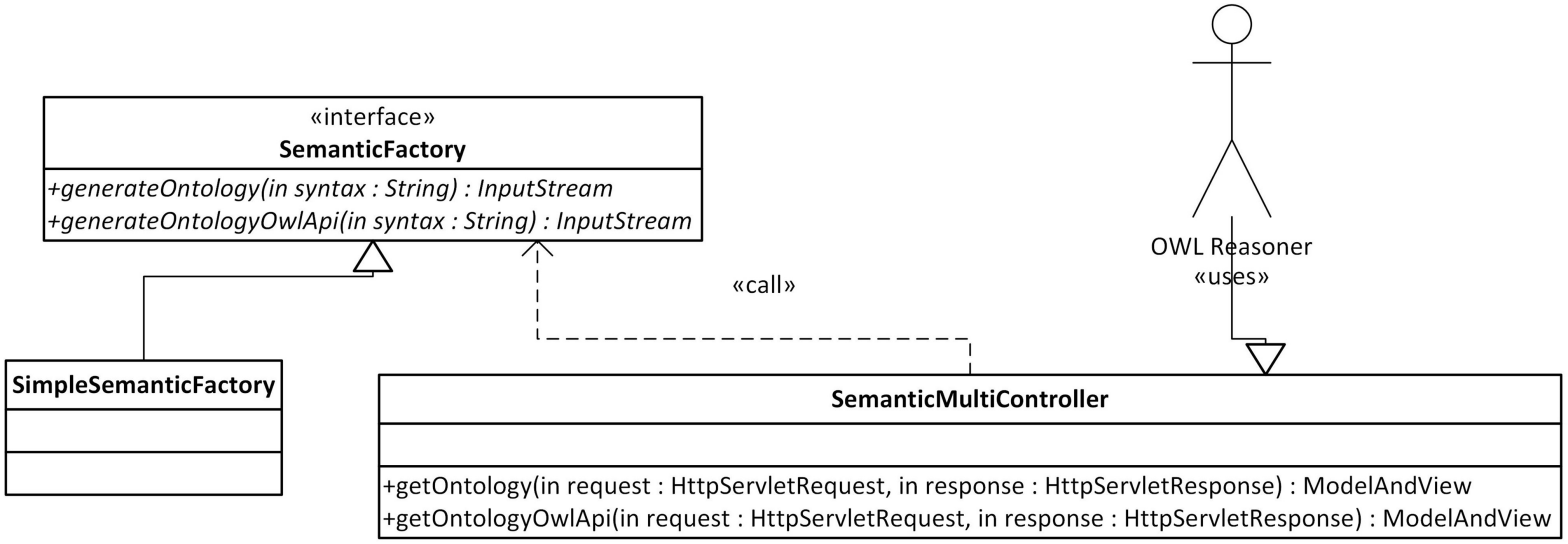
**Semantic Framework Integration.** A user (e.g., OWL reasoner) works with the *SemanticMulticontroller* interface. This controller has two methods, *getOntology* and *getOntologyOwlApi*. A required output syntax is passed to the methods as a part of the HttpServletRequest parameter. The Semantic Framework is called within the methods of the implemented *SemanticFactory* Interface.

**Figure 6:**
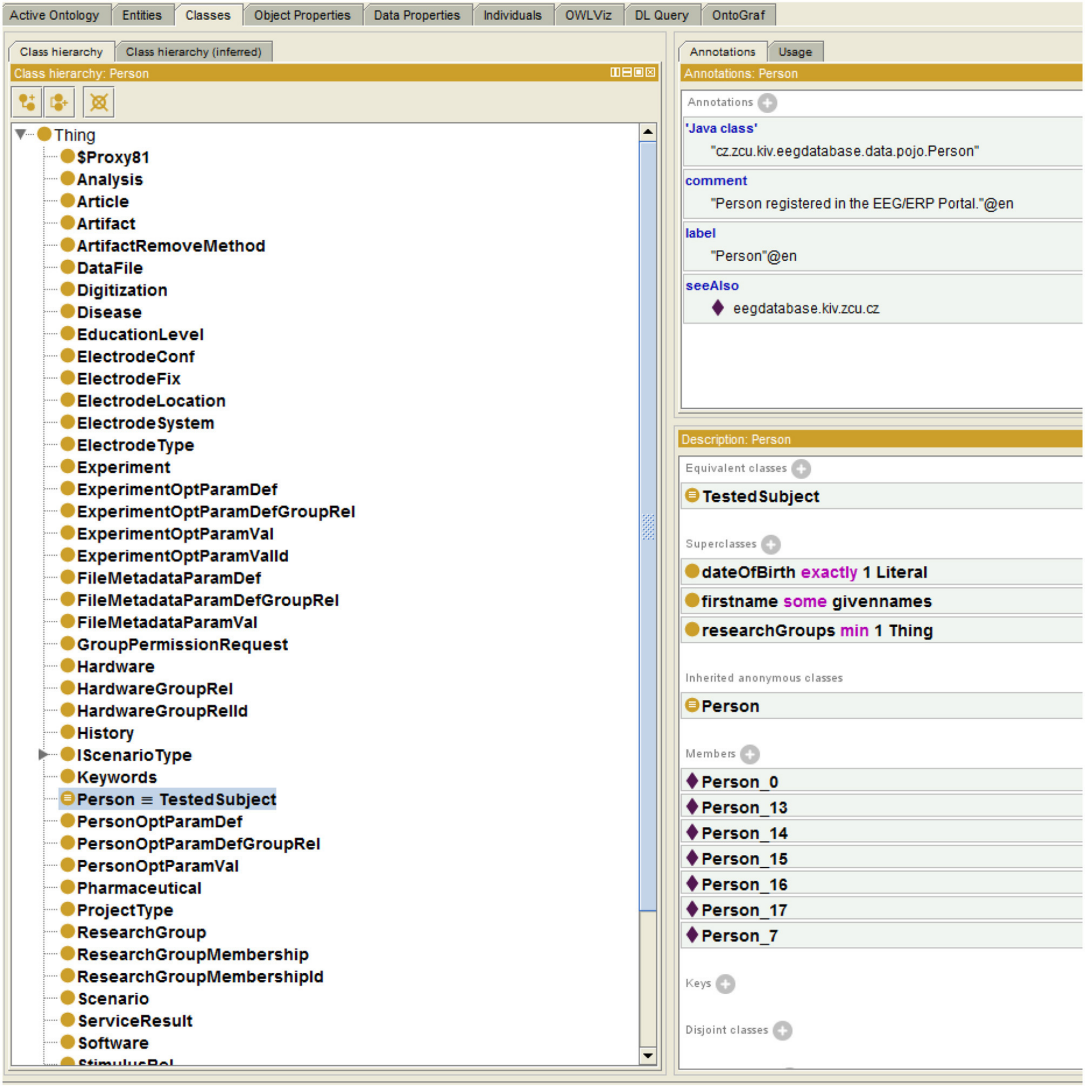
**Protége Visualization.** The left column shows ontology classes. All classes are subclasses of a superclass *Thing*. The right window shows the class *Person*. The class descriptions (*EquivalentClass*) and properties (e.g., *researchGroups* - each person is a member of at least one research group) are transformed from the enriched JavaBean (see Listing 1.3). The class instances (e.g., Person_0) are originally obtained from the relational database.

The Semantic Framework was integrated into the EEG/ERP Portal (Figure 4). The internal logic^4^ calls a Semantic Framework API (the UML diagram describing the Semantic module API in the EEG/ERP Portal is shown in Figure 5) using a built-in timer at regular intervals. The created ontology document is stored in a temporary file that is further serialized into a required syntax. Syntaxes *RDF/XML*, *OWL/XML*, *RDF/XML-ABBREV*, *N-TRIPLE*, *TURTLE*, *N3*, *N3-PP*, *N3-PLAIN*, and *N3-TRIPLE* are currently supported. The *SemanticMultiController* is listening on a specific URL with the *GET* parameter. For instance, when a reasoner visits the URL http://eegdatabase.kiv.zcu.cz/semantic/getOntology.html?type=turtle, it obtains the OWL document in the *turtle* syntax. The output ontology document is valid according to W3C specification. It is formally proved by its visualization in Protége (shown in Figure 6).

**Figure 7:**
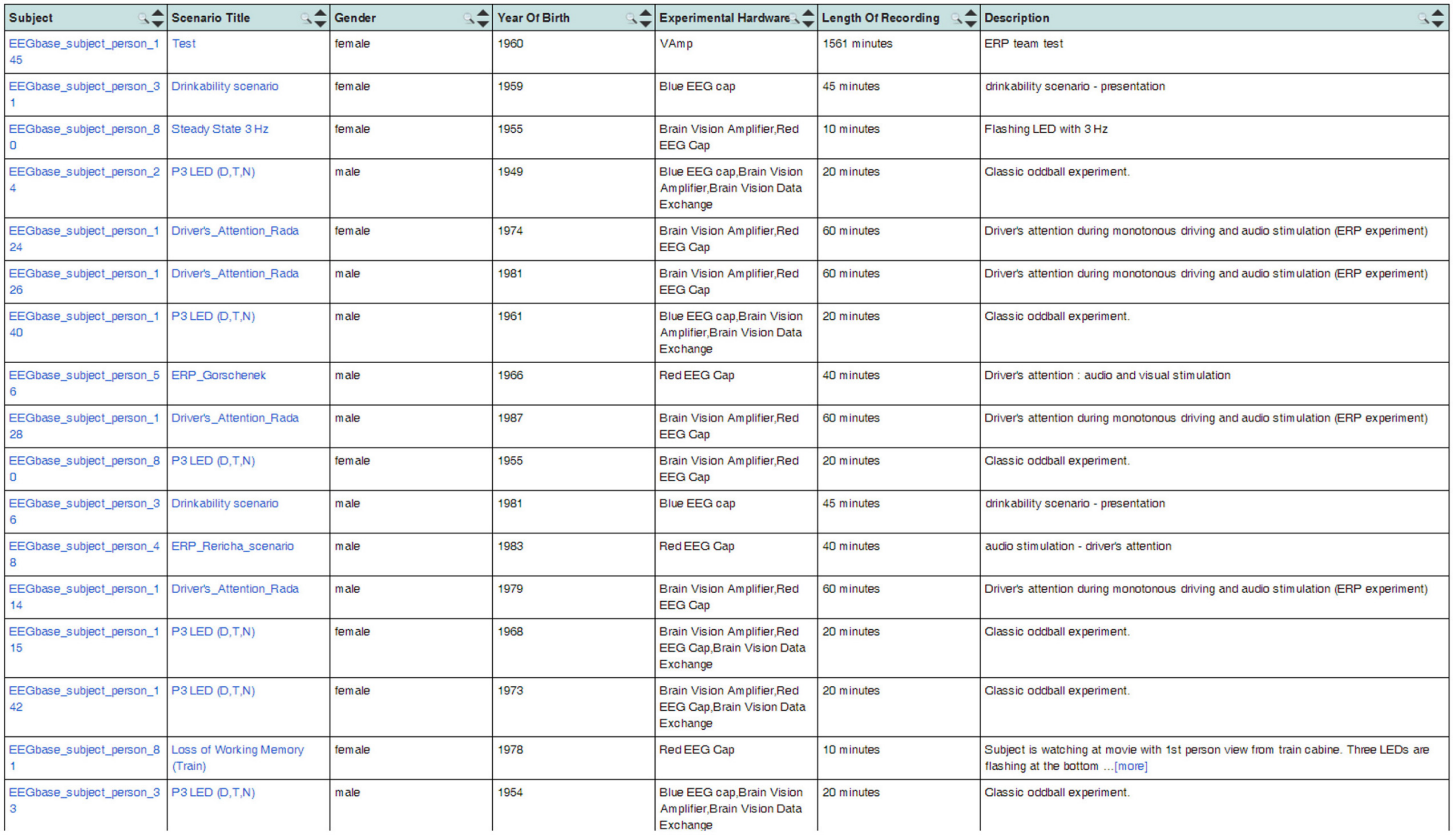
**EEG/ERP Portal in the NIF Registry.** The list of experiments stored in the EEG/ERP Portal is shown in the NIF registry at link https://www.neuinfo.org/mynif/search.php?q=eegbase&t=indexable&list=cover&nif=nif-0000-08190-1. The list contains direct hyperlinks to the experiments stored in the EEG/ERP Portal. When a user clicks on a hyperlink, he/she is redirected to the EEG/ERP Portal.

The generated OWL documents are typically used when registering the EEG/ERP Portal with other providers of neuroinformatics services. We successfully used the Neuroscience Informational Framework (NIF) (Gardner et al., 2008). The NIF framework provides a unified interface for accessing neurophysiological data through resources described by ontology web languages (Gupta et al., 2008). NIF uses a proprietary framework *DISCO* (Marenco et al., 2010). It is an XML-based script containing a static description of the registered resource. The dynamic content is accessed through a generated ontology. The structure of metadata instances is stored in an *Interoperability XML* file that is a part of the DISCO protocol. The interoperability file is stored in the root directory of the EEG/ERP Portal together with generated DISCO files. The NIF framework reloads it at regular intervals. It enables dynamic access to the content of the EEG/ERP Portal. Figure 7 shows experiments listed through the NIF registry. Currently more then 100 experiments are available in the NIF registry and new ones are being gradually added.

## 4. Discussion

This article described the possible approaches to the semantic enrichment of structured electrophysiology data. Different views of software engineering and knowledge representation communities on data modeling, reasonable range of semantic descriptions, and used languages and technologies were briefly introduced. The Semantic Web has become (after 13 years of its existence) a kind of connection between these communities. Currently, the real benefits of the Semantic Web can be found in the concept of linked data that is technologically well-supported. However, in general the Semantic Web technologies are not mature, they are often computationally demanding and the community of developers and administrators who develop/maintain/administrate them is significantly smaller then communities interested in “conventional” programming languages and tools. On the other hand, it is worthwhile to use and promote the Semantic Web languages, standards and technologies that can bring to neuroinformatics applications the opportunity to use higher semantic expressivity.

Based on these assumptions we developed a software prototype, the Semantic Framework library, that connect conventional technologies and programming tools (relational database, domain-independent Java-based systems) with the languages and technologies of the Semantic Web (RDF, OWL, JenaBean, Jena, OWL API). The most important contribution is a transformational mechanism that maps common JavaBeans accessing data stored in a relational database into OWL individuals. In addition, the semantic diversity that exists due to the different semantic expressivity of the object-oriented model and the Semantic Web languages was partly addressed using a custom annotation-based approach. Java annotations are enriched by additional semantic constructions that are also transformed to the resulting OWL ontology document. This approach using annotations replaces the traditional modeling of ontologies in an ontological language. The Semantic Framework was integrated in the EEG/ERP Portal and enabled dynamic generation of the output ontology document. This document is used by the NIF to provide a content of the EEG/ERP Portal through its interface.

Although it is difficult to predict the future development of the Semantic Web, at least it can be expected that new proposals for standards will appear and the software tools that will be developed become more mature and stable. These predictions concerning the future development of the Semantic Web (and standards and tools for semantic descriptions in general) are important for our decisions regarding the development of the EEG/ERP Portal and the Semantic Framework itself.

One of the possible challenges is replacement of the relational database with a NoSQL database for storing experimental metadata. Relational-databases are inflexible when structure modifications are required, while NoSQL databases provide higher scalability and availability because of their free schema. NoSQL databases having key-value organization can easily store RDF triples (Papailiou et al., 2012). There are initiatives, e.g., (Ebel and Hulin, 2012), that investigate the transformation of a common relational database to a NoSQL database. Currently we replaced a part of the relational database for the NoSQL database ElasticSearch.

Another challenge is to integrate a standardized data format and metadata structures into the EEG/ERP Portal. We participate in these standardization activities within INCF Electrophysiology Task Force and within the group developing an experimental ontology for neurophysiology (Ontology for Experimental Neurophysiology Working Group, 2013). Even the partial outcomes of these groups are continuously integrated into the EEG/ERP Portal.

The presented approach was used and tested in the electrophysiology domain, but the mapping mechanism implemented in the Semantic Framework can be easily applied to other domains.

### Conflict of interest statement

The authors declare that the research was conducted in the absence of any commercial or financial relationships that could be construed as a potential conflict of interest.

## Footnotes

^1^http://www.w3.org/

^2^JavaBeans, as reusable components, are named Java classes with class attributes which are accessed only by get/set methods.

^3^The project repository is available at: https://github.com/NEUROINFORMATICS-GROUP-FAV-KIV-ZCU/Semantic-Framework

^4^Spring MVC is used. It practically implements a Model-View-Controller design pattern.
